# A Theoretical and Practical Treatise on Venereal Diseases

**Published:** 1841-10

**Authors:** 


### Art. IV.

1. *Précis Théorique et Pratique sur les Maladies Vénériennes.* Par P. Baumès, Chirurgien en Chef de l'Hospice de l'Antiquaille de Lyon.—*Paris et Lyon*, 1840. 2 vols. pp. 413, 583.

*A Theoretical and Practical Treatise on Venereal Diseases.* By P. Baumes, Senior Surgeon to the Infirmary "de l'Antiquaille" of Lyons.—*Paris and Lyons*, 1840.

2. *A complete Practical Treatise on Venereal Diseases, and their immediate and remote consequences; including observations on certain affections of the uterus, attended with discharges.* By William Acton, late Externe at the Female Venereal Hospital, Paris.—With an Atlas of Plates.—*London*, 1841. 8vo, pp. 410.

Mr. Acton's work is devoted almost exclusively to an exposition of the theory and practice of M. Ricord in reference to venereal diseases; we have therefore thought it right to include under the same notice the treatise of M. Baumès of Lyons, who differs, and we think very justly, from M. Ricord on many points of very great importance. Mr. Acton's book is divided into two parts: the first treating of syphiloid diseases, by which is meant gonorrhœa, its varieties, and consequences; the second including syphilis, properly so called. We do not find in the second division of Mr. Acton's book anything to detain us; and having in a late number, (Br. & For. Med. Rev., No. XX,) entered very fully into the consideration of the opinions of M. Ricord on syphilis, we shall confine this article to some points of a practical character connected with the first, and confessedly the most difficult branch of the subject.

Mr. Acton's division of venereal diseases into the syphiloid and syphilitic is precisely analogous to that of M. Ricord into virulent and non-virulent affections, the second class being truly specific, and capable of propagation by inoculation under the same form, whilst the specific character of the first class is denied by M. Ricord, we think however, in many instances at least, on insufficient grounds.

Mr. Acton includes all the discharges from the female organs under the generic term, first introduced by Swediaur, of Blenorrhagia, and Blenorrhœa. We object to the use of this term, because English readers, more particularly the junior branches, are not familiar with it, and because we are at a loss to know what precise form of discharge the term is used to designate. Any confusion likely to arise from the use of the word blenorrhagia has been still further increased by Mr. Acton, who, although including all discharges from the female organs under this term, yet speaks of the same disease, when communicated to the male under the title of gonorrhœa: "The next form of blenorrhagia I am about to describe, as it exists in the male, is gonorrhœa." (p. 72.)

Blenorrhagia, by which expression, we must again repeat, Mr. Acton designates *all* discharges from the female organs, is thus defined:

"A simple inflammation of the mucous membrane, a consequence more or less direct of sexual intercourse, not necessarily, though often contagious; this last character depending upon a morbid secretion of the stimulating matter, which acting on another mucous membrane will occasion a blenorrhagia, but will (on inoculation) produce no disease of the cellular tissue into which it is introduced. In fine, blenorrhagia differs in no respect from other inflammations of mucous membranes otherwise than in its usual situation, and in the manner in which it is contracted." (p. 24.)

This most untenable theory, contradicted by every day's experience, and even by the results of inoculation itself, is the reiteration of one lately promulgated by M. Ricord in his notes to Richelot's translation of Hunter. M. Ricord has assumed, and his pupil Mr. Acton has taken up his theory we think without sufficient examination, that all the discharges from the female of a purulent or muco-purulent character, upon whatever dependent and however named, are capable of producing in the male that species of affection known to English surgeons by the name of gonorrhœa. Under this view simple leucorrhœa, or the secretions attendent on simple inflammatory states of the vagina or neck of the uterus, may occasion gonorrhœa in the male from sexual intercourse. Against these wild theories M. Baumès in the volumes before us has raised his voice; and, in a long chapter headed "on the contagious principle of gonorrhœa and its effects," has laboured to prove what English surgeons have never doubted, namely, that pure gonorrhœa is a specific disease. We think M. Baumès has made out a good case, and very satisfactorily established that a pure gonorrhœa is as much a specific disease as a primary venereal sore, giving the characteristic pustule of chancre when tested by inoculation.

We do not mean to deny that discharges from the male urethra, the result of sexual intercourse, may be produced by many other causes than the contagion of gonorrhœa, and even by many other species of discharge on the part of the female.

"Doubtless sexual intercourse with a female during the menstrual period, (more particularly if the discharge at these periods be of an unusually irritating character) or with one affected with uterine or vaginal discharges of a more or less acrid nature, which reddens and excoriates the vulva or thighs of the female, may produce urethral discharges in the male, but how different are these in duration, intensity, and consequences from a pure gonorrhœa." (Baumès, vol. i. p. 197.)

Benjamin Bell had long ago distinguished these two species of gonorrhœa in the male, under the terms of gonorrhœa simplex and gonorrhœa virulenta.

Again, these simple gonorrhœas are not capable of propagation in the same manner that a pure gonorrhœa generally is; in fact they are not contagious. M. Baumès has collected a number of very valuable cases on the effects of these two forms of discharge, which will well repay an attentive perusal. Most writers have endeavoured to discover some distinguishing mark between gonorrhœa and other discharges from the vagina, which we may call leucorrhœal. Hunter, Clarke, Churchill, and others have failed in doing so, and it does not appear that the speculum has as yet thrown any additional light on the subject. That differences do exist in the nature of these discharges on the part of the female we are perfectly convinced; differences which the speculum cannot distinguish, but which are evident in their effects upon the male; they are dissimilar in their duration, intensity, consequences; and, still farther, the two are not to be cured by the same remedies.

We will put a case which constantly occurs in practice, and of which, in an experience pretty much extended, we have seen many: M. Baumès also gives several of the same character. A person has connexion with his wife during the time that she is suffering from heat and swelling of the labia, accompanied with a discharge of a mucous or even muco-purulent character, the connexion is repeated time after time without any ill consequences to the husband; after a debauch the connexion is again repeated, and the husband perceives a discharge from the urethra, accompanied by slight scalding in making water, he may conclude himself diseased, and under such an impression vast anxiety may be occasioned. Let him, however, take a smart aperient, and live low for a few days, at the same time using perhaps a mild astringent injection, and his disease shortly disappears. This is simple gonorrhœa, and may be produced by a great variety of causes. On the other hand, the female being perfectly well, the husband goes astray, and has connexion with what M. Baumès would call "an intercurrent mistress," in fact a woman of the town. In a few days he is seized with a discharge from the urethra, accompanied by a greater or less degree of active inflammation; he has intense pain in passing his urine, the discharge goes on little influenced by remedies, he gets in the third week a swelled testicle, the glands in the groin enlarge, and perhaps suppurate, and ultimately the disease terminates in gleet, and stricture. This is a pure and specific gonorrhœa, and a totally different disease from the one at first described, being in our opinion, and in that of English surgeons generally, specific in its character, to be cured by specific remedies, and always propagated under the same forms when the matter of the discharge is applied to a mucous surface.

"Between the muco-pus of a pure gonorrhœa, and the pus or muco-pus of other discharges there is a difference precisely similar to that which exists between the pus of a chancre producing a characteristic pustule by inoculation, and the pus of other sores the result of sexual intercourse, which do not give this result although no chemical or physical circumstances are capable of showing in what this difference consists." (Baumès, vol. i. p. 208.)

These differences are only to be seized in the effects which the different discharges produce.

*Of the Causes of Blenorrhagia.* Under this head Mr. Acton has some important facts.

Infants are more disposed to the affection than adults; this appearing to depend on the delicate state of their mucous membranes.

Females are much more disposed to discharges of a blenorrhagic character than males. "In Paris," says M. Ricord, "women may be said to have habitually a discharge, call it what you will, leucorrhœa, gonorrhœa, fluor albus, &c. &c., it affects all ages and stations. Were I called upon," says the same author, "to estimate the proportion of discharges in the male and female, I should say that it is a hundred times greater in the little girl than in the boy; a thousand times greater in the adult female than in the male." (Acton, p. 25.) These facts speak for themselves; they at once show that if these discharges on the part of the female are so universal in the French metropolis, they depend upon simple or constitutional causes, and are very different in their effects from the poison of a pure gonorrhœa.

The lymphatic temperament, residence in a moist climate, a damp situation, spring and autumn, light clothing, and stimulating food are adduced as powerful predisposing causes of blenorrhagia. The presence of foreign bodies in the vagina, the frequent use of enemata, intestinal worms, renal and vesical affections, menstruation, and inattention to cleanliness, are all capable of producing blenorrhagic discharges. They commonly accompany pregnancy, and succeed to labour; and may be produced by certain morbid states of the constitution, as scrofula, gout, various skin diseases, and the secondary forms of syphilis.

"I may observe," says Mr. Acton, "that thus considered blenorrhagia presents nothing that is specific; it may arise under the most varied circumstances and causes; its existence in the male and female, therefore, of itself is no proof of libertinism; it may occur in the most modest as well as the youngest child; hence, in medical jurisprudence the necessity of being guarded in our opinion; and the surgeon, in family disputes on the subject of contagion, should be especially cautious, and always lean to the weak side." (p. 30.)

Here we perfectly agree with Mr. Acton, differing from him, as we have already stated, only in maintaining that there are specific discharges as well as these enumerated, which latter we may say depend on simple and not on specific causes. It is frequently the case that in consequence of family feuds, a female presents herself and asks the surgeon for a certificate to the effect that she is not the subject of any venereal affection. In the uncertainty as to the true character of a discharge, even after examination with the speculum, M. Ricord recommends, in the absence of ulceration, the following form of certificate:

"I certify, &c., that            presents no symptom of a syphilitic disease, but has a catarrh of the vagina, uterus, &c., and may probably (or not, as may be,) communicate the disease to another." (Acton, p. 33.)

*The Pathology of Blenorrhagia in the Female.* In this section Mr. Acton gives the result of his own observations with those of M. Ricord, M. Emery of St. Louis, M. Danyau, surgeon to the Female Venereal Hospital, and M. Vidal de Cassis. According to M. Ricord, "whatever may have been the cause of the discharge, still the urethra, vulva, vagina, or uterus may be alone or simultaneously affected. Nevertheless, M.

Ricord is persuaded that in the female the urethra is more generally affected alone, or at the same time with the other organs of generation, when the blenorrhagia is the result of impure connexion." The same remark had been previously made by Gibert. In some forms of blenorrhagia in the female, the mucous membrane of the vagina, &c., is found in its whole extent of a red colour, at other times the redness occurs in isolated patches, "with swelling, heat, and pain, unattended by any secretion; thus presenting an erysipelatous state, which may last a short time and then disappear." Other cases of this kind give rise to a morbid secretion, the colour and consistence of which are variable. The differences in the characters of the secretions appear to have no reference to the causes which have produced them. The first of these varieties appears to be the pathology of that form of disease which has been particularly described by M. Baumès under the name of "dry gonorrhœa."

"In examining the vulva, vagina, or the neck of the uterus, we have observed the mucous membrane covered with papulæ, or follicles more or less developed, constituting a "papular vaginitis, or utero-vaginitis," *psorélytrie,* as M. Ricord terms it, sometimes assuming the form of small spots, in size not larger than a pin's head, isolated, or more or less confluent. In other cases these papulæ look like granulations deprived of their epithelium; lastly, they may assume a fungous appearance, in the form of vegetations. On the same portions of the mucous membrane we have distinctly seen patches more or less numerous, and varying in extent, which have a striking analogy with the suppurating surface of the skin on which a blister has been applied. M. Ricord has likewise witnessed a case in which an eruption of herpes phlyctenoides was present on the neck of the uterus and the posterior part of the vagina; lastly, we may find ulcerations of every description seated on the whole or any part of the surface of the genito-urinary mucous membrane.

"The discharges from the urethra, vulva, vagina, and uterus, which we have examined have been very various; but the difference has not appeared to us connected with any one lesion or cause more than another. The acute stage, whatever may be the particular lesion, causes at its commencement a secretion almost wholly serous, or only consisting of healthy mucus, more abundant than usual, but becoming opaque, then purulent, or of a darkish yellow colour, sometimes greenish, and at times mixed with blood. The chronic stage often gives rise to a milky secretion of a thickish consistence, similar to that of cheese, or simply to a mucous flux.

"The chronic discharge may put on a rusty appearance, and become tinged with a larger or smaller quantity of blood. These secretions, whether in the acute or chronic stage may have no smell, or on the contrary, have a very unpleasant odour, particularly where the mucous papulæ exist. The smell *sui generis* is often so decided, that it is characteristic in great number of cases, as under other circumstances it resembles the smell of cancer, or of feculent matter. The only differences which result from the particular seat of the blenorrhagia are, that the secretions which come from the uterus are always more mucous, thready, and collected into flocculi; whereas, those which escape from the urethra, vulva, or vagina, present a less tenacious character than the others." (Acton, pp. 172-3.)

These varied appearances are well represented in the first three plates given by Mr. Acton. Of course the true pathology of the membranes is not to be recognized without the use of the speculum, and whatever facility custom or something worse may give the surgeon in the French metropolis, there exists in this country, even in the hospitals, and amongst prostitutes of the lowest grades, a prejudice against the use of this instrument which is not likely suddenly to be overcome. This feeling also appears, from Mr. Acton's own account, to have existed also among the Parisian "filles de joie." "At the hospital where M. Ricord took the service in 1830, a revolution broke out, and a *strike* against the speculum occurred." Bread and water diet was necessary to quell this "revolt of the harem."

So accustomed, however, says Mr. Acton, is the Parisian female now to the use of this instrument, that M. Ricord frequently receives messages from ladies "in high life" (p. 180), requesting him to visit them, and begging he will not forget *to put his speculum in his pocket*. We do not quite understand these penchants of females in high life for speculum examinations when there is little the matter, or rather where the surgeon has to look whether anything is the matter or not. We should be sorry to see the English surgeon on such a footing with the English matron, neither do we believe we are likely to see it.

The discharges from the male urethra are dependent on several morbid conditions existing in the course of the canal. In the first place there may be a mere hypersecretion of mucus, the result of errors or excesses in diet, or dependent upon other causes which we have already passed in review. Again; discharges from the male urethra of a mucous or muco-purulent character occur after connexion with women menstruating, affected with fluor albus, or various forms of irritation of some part of the genito-urinary system accompanied by discharge. These generally soon subside, are not specific; and, following Bell, we term them simple gonorrhœas. The true "gonorrhœa virulenta" of English writers next succeeds. It may be necessary here perhaps to mention that the term virulent gonorrhœa is understood by M. Ricord and his school in a different sense to that in which it is understood by English surgeons and writers generally. Gonorrhœa virulenta, according to M. Ricord, is a discharge from the urethra depending on the existence of a true chancre in the canal, and is therefore not a syphiloid but a syphilitic disease. Mr. Langston Parker has already, in his Modern Treatment of Syphilitic Diseases, given a description of these chancres, which are termed by the French surgeons "chancres larvés." Ulcerations of other characters may exist in the urethra which are not truly syphilitic, but precisely similar in their nature to those which take place on the surface of other mucous membranes, which have been violently or for a long time inflamed. M. Baumès has given some cases of this kind (pp. 238-9); we have, in our own practice, seen similar forms of disease. According to Lallemand semen commonly exists in discharges from the urethra of a chronic character. The microscope can only decide this question by showing to the surgeon the presence of spermatozoa in the secretion thrown out.

"The existence of blood mixed with muco-pus will generally lead the surgeon to expect an ulceration of the canal which he cannot examine; but here there are many sources of error, as blood may be poured out in consequence of excessive inflammation. Usually, however, I have been able to distinguish, or at least to suspect, the existence of a chancre from the appearance of the discharge, when it has a grayish or reddish tint, and is of thin consistence." (Acton, p. 42.)

Some very interesting questions, about which patients are extremely inquisitive, are examined by Mr. Acton and M. Baumès; these are principally the period at which a chronic discharge from the urethra ceases to be contagious, and what are the chances of secondary symptoms succeeding to such affections. Every practical surgeon knows the difficulty which occasionally occurs of entirely removing the chronic discharges which succeed to gonorrhœa; these, however trivial in quantity they may be, being still possessed of their contagious character, are capable of producing an acute gonorrhœa in a healthy female. Where persons wish to marry, it is of the first importance to determine with certainty, if this be possible,—whether these gleets, if we may so term them, be contagious or not. M. Baumès puts the question in this way: "An individual being given, with a discharge from the urethra, a consequence of gonorrhœa, to determine if this discharge is or is not contagious, to decide whether this individual may or may not communicate the disease to a healthy female." (vol. i. p. 286.) The first point to be determined is the character of the discharge; should it appear to the surgeon to be connected with a chancre of the urethra it will be well to test it by inoculation, when, if the characteristic pustule be produced, there cannot exist a doubt of the contagious character of the affection under its worst form. If the discharge be transparent, or merely mucous, both Mr. Acton, M. Ricord, and M. Baumès consider that contagion is not to be dreaded, and the patient, if so disposed, may marry without fear. It is difficult, however, to determine by mere inspection whether the discharge is mucous or muco-purulent; here the microscope will guide us, and "whatever may have been the duration of the complaint," or to whatever amount the discharge may have been reduced, should it contain pus-globules, "it is capable of causing a similar complaint in any mucous membrane with which it comes in contact."

"Should, therefore, any pus be present in the secretion, the surgeon ought never to sanction sexual intercourse; if the secretion be not purulent, let him wait some days before he gives his permission, to see if it does not become so. Impress strongly on your patients the necessity of abstaining from sexual intercourse immediately any pus appears mixed with the secretion. Patients will sometimes ask the following questions: 'I am obliged to have connexion with my husband; now, Doctor, I am suffering under a discharge, what complaint will he contract from me?' Inoculation* will alone answer the question, and the surgeon will at once be able to tell the probable consequences. The same question relates to marriage; and patients (says M. Ricord) present themselves to me to know whether they may marry, for often their fortune may depend upon a marriage. I persuade them against it if they have a simple gonorrhœa [i. e. in our acceptation of the term an ordinary gonorrhœa,] but if it be a virulent complaint [i. e. in the pathology of M. Ricord a chancre in the urethra], I wash my hands completely of the affair; if they still persist, I tell them that they may give a gonorrhœa to their wives, which unless cured previous to confinement, may cause a loss of eyesight to the child. Called afterwards to cure the lady, I attempt to explain the affection which she has contracted by speaking of the fatigue of the honeymoon, as well as the déjeûner à la fourchette, and in the interim cure both parties, of course forbidding connexion." (Acton, pp. 49-51.)

The next question we shall examine from the authors before us is the

* We refer our readers to the article on "Syphilis" in Number XX. of the Review, for a full account of inoculation in reference to venereal sores.

probability of secondary affections succeeding to purely gonorrhœal diseases. Mr. Acton does not speak of any form of secondary affection succeeding to gonorrhœa, adopting, we suppose, the opinions of M. Ricord, that such consequences do not occur. M. Baumès entertains a different opinion. M. Ricord says (Richelot's Translation of Hunter, p. 178), "there does not exist a single authentic fact in the history of science which proves that an individual, in whom the mucous surfaces have been examined during the course of a gonorrhœa, without the complication of chancre, has ever subsequently been the subject of constitutional syphilis." Many modern writers, amongst whom we may mention the late Dr. Wallace, fully believe in the possibility of secondary symptoms succeeding to gonorrhœa; we have before had occasion to state that we believe the discharges from the urethra to be of various kinds, and do not dispute the fact of constitutional syphilis succeeding to some forms of primary disease in the male, characterized by urethral discharge; on the whole, however, we are disposed to adopt M. Ricord's view of the question, believing that a true gonorrhœa is rarely if ever followed by constitutional symptoms. M. Baumès brings forward some cases in which constitutional symptoms, characterized by copper-coloured patches and papulæ, succeeded to balanitis or discharge from the external surface of the glands, and from the prepuce, without ulceration or breach of surface. This form of disease is considered by most modern writers, Ricord, Parker, Acton, &c., as a variety of gonorrhœa, differing from the urethral variety merely in its seat. In the cases mentioned by M. Baumès this external gonorrhœa was followed by the falling off of the hair, and eruptions precisely similar to those which follow primary venereal sores, and were curable only by mercury. In the first case the patient had never before had any venereal affection till he contracted a balanitis, characterized by redness, heat, and itching of the external surface of the glans penis, and neighbouring portion of the prepuce, to which succeeded a purulent discharge "without any kind of excoriation or wound." This was succeeded in four months by copper-coloured patches on the forehead and chest, and a female with whom this patient cohabited became affected with heat and swelling in the genitals, pain in making water, and two months after an eruption on the inside of the thighs, the nose, and the forehead. The female was declared diseased, put on the use of mercury (Sirop de Larrey) with sarsaparilla, and recovered. The first patient, still suffering from the affection which he did not consider syphilitic, took to himself a second mistress, the first having married after her recovery. This second female soon became affected with the same symptoms in the genitals as the first, and two or three months afterwards "constitutional affection" made its appearance which ultimately assumed a pustular form. The prospect of an advantageous marriage presented itself, and our patient now separated from his second mistress and married. In a short time the wife was affected, as her temporary substitutes had been before her, and subsequently with eruptions of a like character. The patient and his wife now put themselves under the care of M. Baumès, who states that the only disease on the genitals with which the husband was affected was redness of the glans penis, with purulent discharge; no ulceration, breach of surface, or trace of cicatrix. They were put upon a mercurial treatment and both perfectly recovered. M. Baumès adduces another case precisely similar to this.

These primary forms of disease, which have been named by foreign writers "balanitis, or external gonorrhœa," have been of late supposed to be precisely identical in their character with that disease when seated in the urethra, differing from it, as we have before said, merely in its seat. We are inclined, however, to believe that in many cases this analogy is not correct, though it may hold good in some, since what appears a simple catarrhal affection in the first instance frequently degenerates into ulcerations more or less extensive; hence Mr. Acton, in quoting the opinions of M. Puch, one of the surgeons to the venereal hospital, states that M. Puch considers that simple balanitis may produce a chancre, and thus induce secondary symptoms. This, however, is contrary to M. Ricord's view and Mr. Acton's. We are convinced that what is termed "balanitis" may arise from several causes, and know from our own experience that what *appears* as mere catarrhal affection may be followed by bubo and secondary symptoms. Dr. Wallace supported the same view. We dwell upon this subject at some length, because of its great practical importance, and more particularly for the purpose of guarding the young surgeon against following implicitly the opinions of M. Ricord, now so fashionable on this matter, since by so doing he might compromise his reputation in explicitly stating that no secondary symptoms would follow what appeared to him a mere external gonorrhœa.

M. Baumès and Mr. Acton divide the treatment of blenorrhagia into three stages,—the preventive, the abortive, and the curative. Under the first head Mr. Acton, in imitation of M. Ricord, speaks in the first place of the precautions to be taken by diseased persons to prevent the communication of the disease to healthy individuals; and secondly, of those to be observed by a sound individual who exposes himself to contagion. For these we refer to the original. The abortive treatment is directed to cut short the disease before it can be completely established. We shall speak of it only in its application to gonorrhœa in the male.

"At the commencement of a discharge from the urethra, and previous to any redness round the orifice, or pain felt in making water, the surgeon will frequently be able at once to cut short the affection and cure his patient; under other circumstances, this plan will not avail. It consists, in addition to the general means spoken of under the head of the abortive treatment of blenorrhagia, (namely, the free internal use of copaiba, cubebs, or turpentine,) in employing during the succeeding forty-eight hours twelve injections of the nitrate of silver at regular intervals: the strength of the solution of the salt should be two grains to eight ounces of distilled water. Let the injections be then left off, and cubebs or copaiba freely given. If the cases be recent, and the disease not too far advanced, this treatment will succeed fifty times in a hundred cases in checking the disease, and there is no fear of occasioning stricture or swelled testicle at this period of the complaint. Under this treatment the running will at once cease, but to complete the cure it will be necessary to continue the cubebs, diminishing gradually the dose. No further recourse should be had to injections, as a continuance in their use would only tend to keep up irritation; at the end of fifteen days the surgeon may allow his patient to resume his usual habits." (Acton, pp. 79-80.)

The injections should be used cold, by means of a glass syringe made for the purpose. According to M. Baumès, it is useless to attempt the abortive treatment at a period later than forty-eight hours from the first appearance of the disease, yet he particularly insists upon the propriety of attempting it in all fit cases, since in many constitutions, as the lymphatic or scrofulous, gonorrhœa, when once established, is so prone to run on to the chronic state in spite of all remedies, and thus to expose the patient to all its attendant evils. M. Baumès thinks that in plethoric subjects this treatment would be more likely to succeed were it preceded by free venesection ; he brings forward some cases where it succeeded well, the disease disappearing on the third day ; these, however, are contrasted with others, where the injections were followed by violent inflammation of the urethra, the disease was prolonged, became rebellious, and very difficult to cure. It is a game, even in M. Ricord's view of the question, of double or quits.

Neither Mr. Acton nor M. Baumès have added anything to our knowledge of the treatment of established gonorrhœa either in the male or female. The means generally noticed by authors are passed in review. In fact, Mr. Acton's list of remedies is rather meager. There are many preparations of copaiba and cubebs very efficacious, to which he has not even alluded. M. Baumès' work has an appendix of formulæ, which is wanting in the treatise of Mr. Acton.

One of the most frequent and troublesome concomitants of a gonorrhœa is what is known by English surgeons under the name of swelled testicle, described by M. Ricord as " blenorrhagic epididymitis." This affection rarely occurs in the first week of a gonorrhœa, and not frequently in the second. From some statistical tables given by M. Gaussaile in the Archives de Médecine, we learn that during the first week of a gonorrhœa, three cases occurred; during the second, four cases ; during the third, five cases ; fourth, sixteen cases ; fifth and sixth, thirty-nine cases ; two months, two cases; three months, one case.

Amongst the predisposing causes of this affection Mr. Acton enumerates fatigue, violent exercise, repeated sexual intercourse, and any circumstance producing excitement of the organs. Various occupations predispose to it, as those of weavers, turners, grooms, and all trades where the testes are exposed to frequent friction. A flaccid state of the scrotum is also to be ranked amongst the predisposing causes : a strong cremaster and firm scrotum is rarely met with in individuals suffering from hernia humoralis.

"The direct cause consists in inflammation of the urethra. Observation shows that this may take place by direct continuation of the inflammation along the vesiculæ seminales and vas deferens to the epididymis; or it would appear, by virtue of a common law common to many mucous surfaces, extremities of canals may become sympathetically affected, the intervening surface not perceptibly participating in the inflammation. Of the fact there can be no doubt, that the epididymis is often affected, the cord remaining free from disease." (Acton, p. 93.)

Neither Mr. Acton nor M. Ricord attribute the occurrence of swelled testicle to the use of injections, or the quick suppression of the discharge, but to the extension of inflammation in the manner already named. The tables of M. Gaussail show, in conformity with the observations of M. Ricord, that the swelled testicle is much more frequent in the fourth and fifth weeks of the disease ; hence the quicker the primary affection is cured, the less chance is there of the testes becoming affected. A curious case, bearing upon the differential diagnosis of this affection, is mentioned by Mr. Acton:

"A young man, twenty-four years of age, was in the habit of amusing himself, when a boy, by pushing his testicles into the abdomen. Two months previous to his admission into the Hôpital du Midi, he contracted a gonorrhœa which discharged profusely; he continued, notwithstanding, his employment, (that of a wheelwright), a business which requires great bodily exertion: in about a fortnight after he felt a painful sensation in the left groin, or a colic, as he expressed himself, in the loins; and this becoming worse he entered the hospital a month after the commencement of his complaint, suffering under great pain in the inguinal region, which was greatly inflamed, whilst pressure on this part produced that peculiar feeling, but in a greater degree, which is excited when the testicle itself is compressed. On examining this patient, no testis was found on the left side of the scrotum; but, on passing the finger into the left inguinal canal, a rounded body was distinctly felt, resembling the testis in shape, and the patient stated that he experienced a similar kind of pain to that felt when the testicle on the opposite side was squeezed." (p. 95.)

The case was recognized as one of hernia humoralis, notwithstanding the unnatural position of the testes. It is not improbable that such a case might be mistaken for a strangulated inguinal hernia, more particularly when such symptoms as vomiting, constipation, and tenderness of the abdomen are present. The history of the concomitant affection, and the absence of the testes in the scrotum, are the chief points which would decide the surgeon. Should such a case coexist with a strangulated hernia, it would form a curious and puzzling complication, and one which would render the operation extremely embarrassing.

There are few cases on record illustrating the morbid anatomy of this affection, since it is one which is rarely fatal. Mr. Acton has, however, collected a few, where patients have died during the course of such an affection from other diseases. "In a simple case which M. Ricord presented to the Academy of Medicine, the epididymis was affected alone. In another case where severer symptoms had been observed, the tunica vaginalis presented traces of pus and false membrane. In the most severe forms lymph was effused amongst the seminiferous vessels." In two cases observed by M. Gaussail, and recorded in the Archives de Médecine, the following appearances were noted:

"CASE I. The epididymis was double the usual size, and hard. The testis of the same size appeared to present twice its ordinary volume; this however was found to depend in great part upon an accumulation of a thick turbid serum, somewhat bloody, which flowed out when the tunica vaginalis was opened. The tunica albuginea seemed thicker than usual; its surface presented a large number of minute vessels spreading out in various directions on its surface. The substance of the testis presented no appreciable change, its consistence was somewhat firmer than usual, and the colour deeper.

"CASE II. The vesiculæ seminales larger than usual, and firmer to the finger; on the left side they were much injected, and of a dark red colour, containing a large quantity of a yellowish white substance, which was somewhat granular. Both the vasa differentia presented similar traces of inflammation, and were filled with the same matter. The epididymis of either side was voluminous; the surface resembled the colour of lees of wine, but this discoloration did not extend to the testis. The testicles had their ordinary volume, some vessels were observed ramifying on there surface. A small quantity of reddish serum was found in the tunica vaginalis." (Acton, p. 91.)

The prognosis of this disease, although generally favorable, is not always so. It may terminate in suppuration of the testicle, an event however but very rarely happening, in hydrocele or chronic disease of the substance of the testis itself. We believe the latter consequence to succeed much more frequently to purely syphilitic diseases, than to gonorrhœal affections. We do not agree with Mr. Acton that hydrocele rarely succeeds to hernia humoralis, or does not require more than a palliative treatment. We have seen numerous instances where hydrocele has complicated the affection, with apparent enlargement of the testicle itself, if we may judge from the greatly increased weight of the organ. This species of hydrocele certainly does not often require the treatment by operation. We have found mercury pushed to salivation of great service in these consecutive affections of the testicle and its envelopes.

It would be useless to revert here to the treatment of hernia humoralis by compression originally proposed by Fricke of Hamburg, modified by Ricord, and advocated by several modern writers, amongst whom is Mr. Acton. We can only again repeat, from extensive experience of its utility, its vast superiority over all other methods of treatment of the primary forms of orchitis, succeeding to gonorrhœa. A full account of the method of performing it will be found in our Twentieth Number.

Before closing this article we would say a few words on inoculation in reference to primary venereal sores, and just enquire what is the position on which inoculation stands in reference to the therapeutics of primary syphilis. Inoculation pushed to its greatest extent by M. Ricord, has proved beyond a doubt the fact which English surgeons had always acted upon, though we must admit not with so good grounds as they do now, that syphilis is a specific disease, and not the result of the ordinary forms of irritation : M. Ricord has proved that certain sores when tested by inoculation produce a pustule running through certain stages, and terminating in a specific ulcer, capable of being propagated ad infinitum by the same means. M. Ricord however, has only shown that certain sores produce a characteristic pustule by inoculation, and therefore should be those only which are truly syphilitic, yet we find other sores, the result of sexual intercourse, producing secondary symptoms of the worst kind ; and also yielding magically in some instances to mercury where all other remedies had failed, the old Hunterian test of true syphilis.

In the present state of science all we can say is, that certain sores the result of sexual intercourse, and not distinguishable by their external characters from other sores equally produced by sexual intercourse, yield a characteristic pustule by inoculation ; but the sores which do not yield the characteristic pustule are equally liable to be followed by secondary symptoms, and are equally benefited under many circumstances by mercury; in fact with regard to the latter point, the external character of the sore has by almost universal consent been judged the criterion for the necessity of mercury.

We would now say a few words respectively on the merits and execution of the two works before us. Mr. Acton's work is the completest exposition of the opinions and practice of M. Ricord, which has yet been given in this country : Mr. Acton has neglected the whole of the other French writers, and followed M. Ricord alone, and so closely that he has advocated many of what we consider to be the errors of the French surgeon, and which we believe the author, when he becomes familiar with English practice, will renounce. We regret this extreme exclusiveness of doctrine, more particularly as far as the first division of the work is concerned, since it lessens its value otherwise considerable. The first part of the treatise, that devoted to the catarrhal forms of disease, is the one which to the English surgeon will present the greatest degree of novelty, and this from the circumstance already mentioned of the greater facility with which the speculum can be employed in France. The volume of letter-press is accompanied by a folio atlas of plates, representing each of the forms of the syphiloid and syphilitic diseases both in the male and female, and in their primary, secondary, and tertiary stages. These plates are extremely well executed, and add much to the value of the work.

The work of M. Baumès is divided into two parts, the first theoretical, the second practical. In the first division M. Baumès treats of the syphilitic virus, and the contagious principle of gonorrhœa: here, as we have already shown, he is at issue with M. Ricord. The first part also considers the primitive and constitutional effects of this virus and contagious principle, and the general principles of treatment. The second volume contains the particular history and treatment of each form of disease, with an appendix of formulæ. This part although complete, and well and clearly arranged, contains little that the practical surgeon is not already familiar with. The first part is the most valuable, and is directed principally against some of the theories of M. Ricord, which the author controverts by powerful reasoning, and an appeal to well-established facts, drawn not only from his own observation, but from that of some of the principal surgeons in Europe.

---

